# Generating real-world evidence compatible with evidence from randomized controlled trials: a novel observational study design applicable to surgical transfusion research

**DOI:** 10.1186/s12874-022-01787-3

**Published:** 2022-12-06

**Authors:** Xiaochu Yu, Zixing Wang, Lei Wang, Yuguang Huang, Yipeng Wang, Shijie Xin, Guanghua Lei, Shengxiu Zhao, Yali Chen, Xiaobo Guo, Wei Han, Xuerong Yu, Fang Xue, Peng Wu, Wentao Gu, Jingmei Jiang

**Affiliations:** 1 Nephrology Department, Peking Union Medical College Hospital, Chinese Academy of Medical Sciences, Beijing, China; 2 Department of Epidemiology and Biostatistics, Institute of Basic Medical Sciences, Chinese Academy of Medical Sciences/School of Basic Medicine, Peking Union Medical College, Beijing, China; 3 Anaesthesiology Department, Peking Union Medical College Hospital, Chinese Academy of Medical Sciences, Beijing, China; 4 Orthopaedics Department, Peking Union Medical College Hospital, Chinese Academy of Medical Sciences, Beijing, China; 5 Vascular and Thyroid Surgery Department, the First Hospital of China Medical University, Shenyang, China; 6 Orthopaedics Department, Xiangya Hospital, Central South University, Changsha, China; 7 Medical Affairs Department, Qinghai People’s Hospital, Xining, China

**Keywords:** Study design, Bias by indication, Propensity score, Transfusion, Patient outcome

## Abstract

**Background:**

Numerous observational studies have revealed an increased risk of death and complications with transfusion, but this observation has not been confirmed in randomized controlled trials (RCTs). The “transfusion kills patients” paradox persists in real-world observational studies despite application of analytic methods such as propensity-score matching. We propose a new design to address this long-term existing issue, which if left unresolved, will be deleterious to the healthy generation of evidence that supports optimized transfusion practice.

**Methods:**

In the new design, we stress three aspects for reconciling observational studies and RCTs on transfusion safety: (1) re-definition of the study population according to a stable hemoglobin range (gray zone of transfusion decision; 7.5–9.5 g/dL in this study); (2) selection of comparison groups according to a trigger value (last hemoglobin measurement before transfusion; nadir during hospital stay for control); (3) dealing with patient heterogeneity according to standardized mean difference (SMD) values. We applied the new design to hospitalized older patients (aged ≥60 years) undergoing general surgery at four academic/teaching hospitals. Four datasets were analyzed: a base population before (Base Match−) and after (Base Match+) propensity-score matching to simulate previous observational studies; a study population before (Study Match−) and after (Study Match+) propensity-score matching to demonstrate effects of our design.

**Results:**

Of 6141 older patients, 662 (10.78%) were transfused and showed high heterogeneity compared with those not receiving transfusion, particularly regarding preoperative hemoglobin (mean: 11.0 vs. 13.5 g/dL) and intraoperative bleeding (≥500 mL: 37.9% vs. 2.1%). Patient heterogeneity was reduced with the new design; SMD of the two variables was reduced from approximately 100% (Base Match−) to 0% (Study Match+). Transfusion was related to a higher risk of death and complications in Base Match− (odds ratio [OR], 95% confidence interval [CI]: 2.68, 1.86–3.86) and Base Match+ (2.24, 1.43–3.49), but not in Study Match− (0.77, 0.32–1.86) or Study Match+ (0.66, 0.23–1.89).

**Conclusions:**

We show how choice of study population and analysis could affect real-world study findings. Our results following the new design are in accordance with relevant RCTs, highlighting its value in accelerating the pace of transfusion evidence generation and generalization.

**Supplementary Information:**

The online version contains supplementary material available at 10.1186/s12874-022-01787-3.

## Background

Blood is a precious resource widely required across all clinical disciplines. Shortage of blood is a public health issue [1] and numerous efforts have been made to reduce the use of blood [2]. According to a Cochrane review published at the end of 2021 [3], 48 randomized controlled trials (RCTs) have been conducted comparing patient outcomes following a restrictive versus liberal transfusion strategy, in which a low versus high hemoglobin concentration (e.g., 8 g/dL vs. 10 g/dL) was used as a threshold to trigger transfusion, respectively. Compared with the liberal strategy, the restrictive strategy decreased the need for transfusion by 41% and generally did not have an impact on patient mortality (pooled risk ratio: 0.99, 95% confidence interval [CI]: 0.86–1.15) or morbidity, including cardiac events, myocardial infarction, stroke, and pneumonia. The evidence was assessed as high quality [3] and laid a foundation for recent guidelines recommending more restrictive use of blood [4]. However, there remain substantial variations in real-world transfusion practice because of an unmet need for further evidence, slow evidence generation, and limited patient representativeness using RCT design being a bottleneck [5]. For instance, surgical patients are considered a distinct population that consumes 40–60% of total blood resources [6]; however, until now, related RCTs have been largely limited to orthopedic or cardiac surgery [7].

The past three decades have witnessed a large and growing number of observational studies focused on the safety of transfusion that have evaluated patient outcomes similar to those used in RCTs. Surprisingly, in observational studies, a higher risk of mortality and morbidity is seen in transfused patients versus those who are not transfused [8]. According to several meta-analyses, this finding is consistent in observational studies across surgical categories and other clinical settings [8–10] but it contradicts evidence from RCTs [8], the gold standard of evidence. A common explanation for this apparent “transfusion kills patients” paradox is that patients with anemia or bleeding are more likely to experience unfavorable outcomes [11]. Such “indications” have confounded the actual association between transfusion and outcomes. This theoretical argument of bias by indication, an epidemiological concept of confounding, provides a balance between clinical knowledge and academic interpretations and has thus accelerated the application of advanced confounding-control analysis techniques (e.g., propensity-score matching) in observational studies [12–14]. Unfortunately, confusing results persist in recent publications despite these efforts, and many have begun to question whether observational studies should inform transfusion practice [8]. If this question remains unresolved, it will be deleterious to the healthy generation of evidence in support of using real-world data to investigate transfusion–outcome associations with greater efficiency and ethical feasibility over data from RCTs.

Our team has worked on this issue for 4 years [15, 16]. In 2021, we reported an observational study that replicated and generalized RCT evidence supportive of restrictive transfusion (from orthopedic and cardiac surgery to six surgical specialties) [16]. In the present study, we formalized an observational study design (hereafter termed a hemoglobin-based design) and applied it to older surgical patients, a fragile population with a high demand for transfusion. It is interesting to note that a few RCTs showed better outcomes in this particular population following liberal versus restrictive transfusion [17–19], a finding that challenges both previous observational studies and RCTs [20]. We examined whether such a novel finding could be replicated and augmented by our observational study design using real-world observational data. Via this example study, we showcase the value of the proposed new study design.

## Methods

### New design

By proposing the present hemoglobin-based design, we aim to reconcile observational studies and RCTs investigating transfusion safety. There are three core components of the new design that distinguish it from those of existing studies.

1. Re-definition of the study population.

It is very common for transfusion studies (in both previous observational studies and RCTs) to limit the study population to a specific surgical procedure or disease [7, 21], in addition to other inclusion/exclusion criteria, such as age limits. Rather than following this common practice, we redefined the study population according to a stable hemoglobin range. “Stable” here means no active bleeding, and the range of hemoglobin concentration is determined by the transfusion threshold of interest, e.g., 7.5–9.5 g/dL in our example study. Using these criteria, we focused on the transfusion effect for patients with anemia that are within a gray zone of transfusion decision, according to current guidelines [22]. Data of any patients within this defined range who also meet the study-specific inclusion criteria could be subsequently analyzed.

#### Key point 1

An underlying assumption of the hemoglobin-based definition is that the included patients are a *homogeneous population* regarding the decision for transfusion. The reason is that these patients have a similar level of anemia, despite different surgical categories or other patient-specific conditions. The new definition of study population largely retains the authenticity of real-world data in representing patients with anemia seen in daily practice and accords our design the potential to augment external validity beyond than that of RCTs, which typically have a very narrow patient spectrum.

#### Key point 2

The new definition of study population naturally excludes patients with active bleeding and severe anemia, two strong indications for which non-transfusion is unexplained (but that exist in real-world practice); the new definition excludes unreasonable transfusion beyond the current clinical standard (hemoglobin ≥10 g/dL) [22]. These properties largely avoid bias by indication, which is present in observational studies that attempt to associate transfusion with outcomes using any available sample.

(2) Selection of comparison groups.

In our new design, we selected two comparison groups: a transfused group (exposure) and non-transfused group (control), both defined according to the hemoglobin concentration of interest, hereafter referred to as the *trigger value*. The trigger value is a very important component of the RCT design. For instance, the decision to transfuse a patient is made based on whether the hemoglobin level is below 10 g/dL for the liberal transfusion arm and below 8 g/dL for the restrictive transfusion arm [23]. Similarly, in observational studies, this decision is also primarily based on the hemoglobin level [24]. Because patients can have multiple hemoglobin tests and multiple transfusions in practice, we defined the trigger value as the last measurement before the initial transfusion in the exposure group and the nadir during the hospital stay in the control group. The purpose of these choices is to unify the decision to transfuse (or not transfuse) a patient according to the same decision criterion, namely, the degree of anemia in the patient.

#### Key point 3

**Fig. 1:**
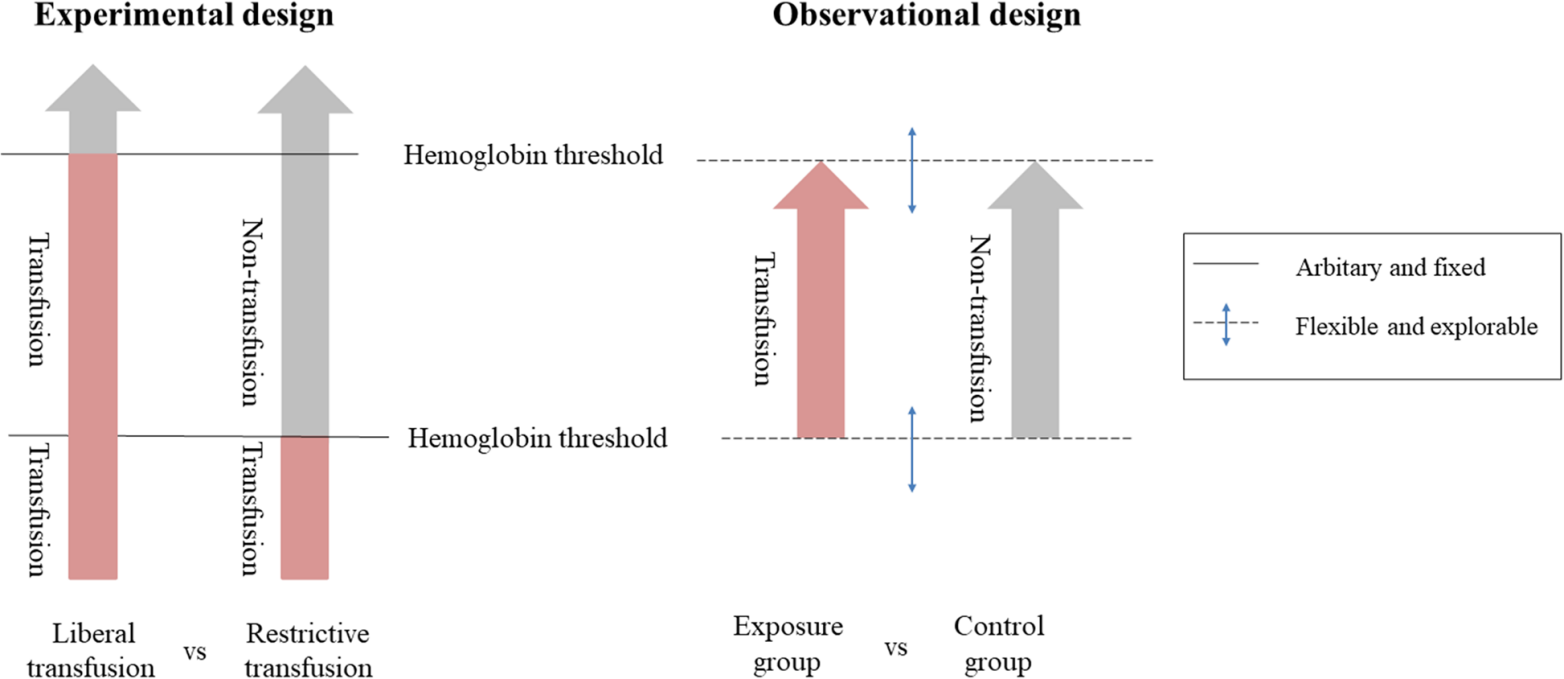
Experimental and observational study designs to investigate transfusion threshold

The comparison of liberal versus restrictive transfusion strategies, in essence, compares the effect of transfusion to that of no transfusion when the anemia level of a patient is within the *critical range* of the transfusion decision, that is, the hemoglobin range defined by the low and high thresholds (see Fig. 1). By targeting the critical range, our new design can approximate the experimental design and the study efficiency is greater in terms of outcome comparison because in the experimental design, the interventions are identical for liberal and restrictive strategies beyond this critical range [3].

(3) Dealing with patient heterogeneity.

Unlike RCTs where the only apparent difference between randomized patient groups is the pre-specified transfusion protocols (liberal or restrictive), patient heterogeneity (i.e., skewness in baseline characteristics between comparison groups) is high in observational studies and is usually not fully recognized nor treated, thereby undermining the validity of effect estimation. To quantify heterogeneity between the comparison groups with observed information, we propose using a uniform measurement, the standardized mean difference (SMD) [25], defined as:

1 $$\left\{\begin{array}{c} SMD=\frac{\left|{\overline{x}}_1-{\overline{x}}_2\right|}{\sqrt{\left({s}_1^2+{s}_2^2\right)/2}}\times 100\%,\kern0.5em \textrm{for}\ \textrm{continous}\ \textrm{variables}\\ {} SMD=\frac{\left|{p}_1-{p}_2\right|}{\sqrt{\left({p}_1\left(1-{p}_1\right)+{p}_2\left(1-{p}_2\right)\right)/2}}\times 100\%,\kern0.5em \textrm{for}\ \textrm{binary}\ \textrm{variables}\end{array}\right.$$

where $\overline{x}$, *s*^2^, and *p* are the mean, variance, and proportion in a comparison group, respectively. Commonly, an SMD value smaller than 10% is suggestive of minor differences between comparison groups.

By investigating apparent, moderate, and minor sources of patient heterogeneity, pertinent approaches such as restriction, stratification, or other statistical methods can then be used to address bias by indication (e.g., severe anemia, bleeding) or other common confounding factors (e.g., age, comorbidity). A continuous effort to monitor and reduce patient heterogeneity in different analytic datasets can help to improve the validity of the estimated transfusion effect, as illustrated in the example below.

### Data source and patient selection

We used real-world data prospectively collected in a multicenter quality improvement project conducted during 2015 to 2016 at four academic/teaching hospitals that represent the regional diversity of China [26]. We focused on hospitalized older patients (aged 60 years and over) undergoing general surgery. This surgical population underwent different categories of procedures (mainly including intestine, gallbladder, thyroid, and stomach surgeries), comprising 51% of the non-orthopedic, non-cardiac surgery volume, and accounting for 55% of red blood cell transfusion among non-orthopedic, non-cardiac surgical patients.

We selected a “base population” to simulate previous observational studies; from this, we further derived a “study population” to demonstrate the effect of the hemoglobin-based design. The criteria for the base population were: (1) major surgery, defined as requiring the presence of an anesthesiologist during surgery; and (2) hospital stay ≥24 hours. In addition to these criteria, the study population was defined as: (3) no bleeding ≥500 mL; and (4) within a hemoglobin range of 7.5–9.5 g/dL. The choice of hemoglobin thresholds for defining the critical range was based on a planned RCT on liberal versus restrictive transfusion among older non-cardiac surgical patients because no pertinent evidence for general surgery patients is available. Patients residing at altitudes of 2000–5000 m above sea level were excluded because the common transfusion threshold may not be applicable. Ethical approval was obtained from the institutional review board of Peking Union Medical College Hospital (approval no.: S-574); requirement for written informed consent was waived because individual information was analyzed anonymously.

### Study variables

For comparability, we selected patient outcomes that are similar to those used in previous observational studies and RCTs [27, 28]; these outcomes were death (in-hospital or within 30 days of discharge) and in-hospital complications, including ischemic events (myocardial infarction, stroke, and acute renal failure); infection (surgical site infection, pneumonia, sepsis, septic shock, and urinary tract infection); and others (cardiac arrest requiring cardiopulmonary resuscitation, heart failure, reintubation, mechanical ventilation for ≥48 hours post-operatively, atelectasis, respiratory failure, wound dehiscence, delayed incision healing, pulmonary embolism, venous thrombosis, and multiple organ dysfunction syndrome). These outcomes are considered to be directly or indirectly associated with anemia. We defined the primary study outcome as a composite of these outcomes to stabilize these low-incident events, i.e., using a binary variable that indicates the occurrence of any of these adverse events.

Transfusion information was obtained directly from clinical blood bank systems. We also considered basic patient information (age, sex, smoking status, body mass index), preoperative comorbidity (hypertension, coronary heart disease, diabetes, stroke, chronic obstructive pulmonary disease), preoperative laboratory test findings (low albumin, high creatinine, and high white blood cell count), and physical status (evaluated by an anesthesiologist and recorded using American Society of Anesthesiologists [ASA] score), intraoperative features (operation time, high blood loss), and postoperative return (intensive care unit [ICU] or other). The data collection methods were standardized according to a study protocol reported elsewhere [26].

### Statistical analysis

To demonstrate the effect of our new design, we analyzed and presented results for four datasets: the base population before (Base Match−) and after (Base Match+) propensity-score matching; and the study population before (Study Match−) and after (Study Match+) propensity-score matching. Propensity scores were calculated with a multivariable logistic regression model using several key covariates, namely, ASA score ≥ 3, preoperative hemoglobin, operation time ≥ 3 hours, blood loss ≥500 mL, and ICU admission for the base population, and ASA score ≥ 3, preoperative hemoglobin, operation time ≥ 3 hours, and ICU admission for the study population; these variables were clinically and/or statistically significantly related to both transfusion and patient outcomes. Matching was based on a 1:1 ratio using the nearest-neighbor method [29]. A caliper of 0.2 standard deviations of the propensity score was used; the choice of caliper value and the selection of key covariates were made considering both the matching rate and balance of covariates between groups [30].

We treated the propensity score (i.e., the estimated individual probability of receiving transfusion) as an overall index of patient heterogeneity and presented the overlapping range between groups using box plots. To closely examine patient heterogeneity, we quantified between-group differences regarding specific variables using the SMD measurement. The study effect of interest (i.e., the transfusion–outcome association) was quantified using odds ratio (OR) estimated in multivariable logistic regression, adjusting for covariates that were significantly related to patient outcomes, namely, ASA score ≥ 3, age ≥ 75 years, preoperative comorbidity, preoperative hemoglobin, operation time ≥ 3 hours, blood loss ≥500 mL, and ICU admission for the base population and ASA score ≥ 3, age ≥ 75 years, preoperative comorbidity, preoperative hemoglobin, and operation time ≥ 3 hours for the study population.

No imputation of missing data was performed because the missing rates are negligible (highest for operation time: 6.86%). A two-tailed *p* value of < 0.05 was considered statistically significant. All analyses were performed using SAS software, version 9.4 (SAS Institute, Cary, NC, USA) and R, version 3.6.3 (The R Foundation for Statistical Computing, Vienna, Austria). Plots were drawn with Python, 3.10.2 (Python Software Foundation, Beaverton, OR, USA).

## Results

### Patient characteristics

A total of 6141 older general surgery patients were included in the base population; the patient characteristics are shown in Table 1. Among them, 662 (10.78%) patients were transfused and showed high heterogeneity compared with patients not receiving transfusion. Specifically, the transfusion group versus the non-transfusion group had a larger proportion of male (61.3% vs. 51.7%) and elderly (≥75 years: 19.2% vs. 15.2%) patients. Although transfused patients had a lower proportion of hypertension (44.0% vs. 50.5%), this group was more likely to have poor physical status (ASA score ≥ 3: 40.5% vs. 22.8%), low albumin (39.9% vs. 12.9%), high creatinine (13.0% vs. 9.5%), and low hemoglobin concentration (median: 11.2 g/dL vs. 13.6 g/dL). In particular, transfused patients had disproportionately higher rates of long-duration surgery (≥3 hours: 65.2% vs. 20.9%), mass bleeding (≥500 mL: 37.9% vs. 2.1%), and admission to the ICU after surgery (22.2% vs. 8.0%). As expected, higher crude rates of complications (15.4% vs. 4.1%) and death (5.9% vs. 0.5%) were observed in the transfused versus non-transfused group, all *p* < 0.05.

**Table 1:** Patient characteristics in the base population

Characteristic	Transfusion	No transfusion	***p***
	(*n* = 662)	(*n* = 5479)	
**Demographics**			
Male, *n* (%)	406 (61.3)	2835 (51.7)	< 0.001
Age > 75, years, *n*(%)	127 (19.2)	831 (15.2)	0.0071
Smoking, *n* (%)	78 (11.8)	547 (10.0)	0.148
BMI, kg/m^2^, mean(SD)	22.57 (3.39)	23.56 (3.46)	< 0.001
**Comorbidity**, *n* (%)			
Hypertension	291 (44.0)	2768 (50.5)	0.0014
Coronary heart disease	70 (10.6)	565 (10.3)	0.834
Diabetes	84 (12.7)	693 (12.6)	0.976
Stroke	5 (0.8)	84 (1.5)	0.114
COPD	8 (1.2)	55 (1.0)	0.621
**ASA score ≥ 3, *n *(%)**	268 (40.5)	1250 (22.8)	< 0.0001
**Preoperative laboratory test**			
Albumin < 35 g/L, *n* (%)	264 (39.9)	707 (12.9)	< 0.0001
High creatinine^a^, *n* (%)	86 (13.0)	523 (9.5)	0.0051
WBC count > 10 × 10^9^/L, *n* (%)	76 (11.5)	483 (8.8)	0.0244
Hemoglobin, g/dL, mean(SD)	11.0 (29.7)	13.5 (22.55)	< 0.001
**Intraoperative feature**, *n* (%)			
Operation time ≥ 3 h	408 (65.2)	1068 (20.9)	< 0.0001
Bleeding volume ≥ 500 mL	251 (37.9)	115 (2.1)	< 0.0001
**Postoperative feature**, *n* (%)			
ICU admission after surgery	147 (22.2)	436 (8.0)	< 0.0001
**Postoperative outcome**, *n* (%)			
Complications	102 (15.41)	226 (4.12)	< 0.0001
Ischemic events	22 (3.32)	26 (0.47)	< 0.0001
Infection	76 (11.48)	170 (3.10)	< 0.0001
Others	19 (2.87)	48 (0.88)	< 0.0001
Death	39 (5.89)	29 (0.53)	< 0.0001

*BMI* body mass index, *WBC* white blood cell, *ASA* American Society of Anesthesiologists, *ICU* intensive care unit, *IQR* interquartile range, *COPD* chronic obstructive pulmonary disease

^a^High creatinine: > 84 μmol/L in women, > 104 μmol/L in men

### Reduction in patient heterogeneity

**Fig. 2:**
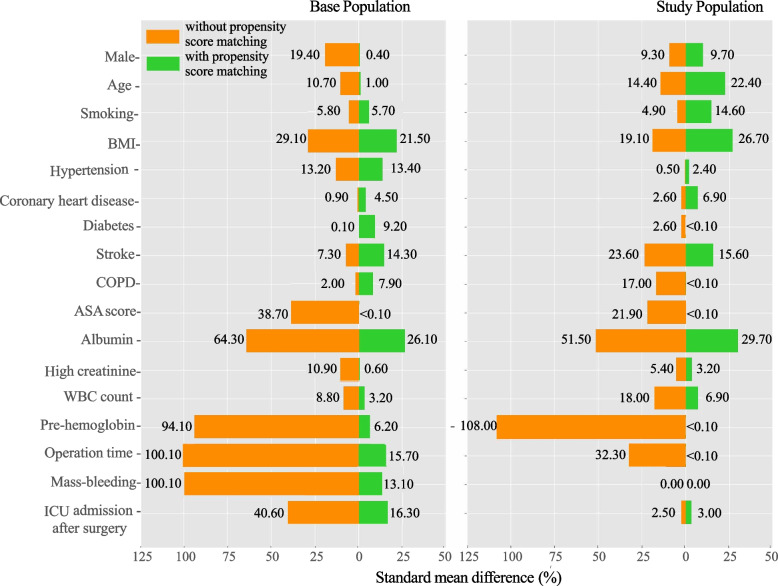
Standard mean difference of covariates. BMI, body mass index; COPD, chronic obstructive pulmonary disease; ICU, intensive care unit; WBC, white blood cell

From the base population (i.e., Base Match−) three datasets were obtained: Base Match+ (*n* = 958; matching rate: 72.4% vs. 8.7% in the exposure vs. control groups), Study Match− (*n* = 715), and Study Match+ (*n* = 164; matching rate: 97.7% vs. 13.3% in the exposure vs. control groups). In Fig. 2, we show how the between-group patient heterogeneity changed along with application of our new definition of the study population and propensity-score matching. Specifically, in Base Match−, the patient covariates had SMD values ranging from 0.1% (for diabetes) to over 100% (for operation time and mass bleeding); by directly applying propensity-score matching (Base Match+), SMD values were significantly decreased but remained above 10% for several covariates, e.g., 13.1% for mass bleeding. In contrast, some covariates naturally became balanced (SMD < 10%) after using the hemoglobin-based new design (Study Match−); the remaining covariates were mostly well balanced after further propensity-score matching (Study Match+). Also note that for both the base and study populations, pre-operation hemoglobin was highly skewed between comparison groups (SMD approximately 100%) unless propensity-score matching was applied (SMD reduced to nearly 0%).

**Fig. 3:**
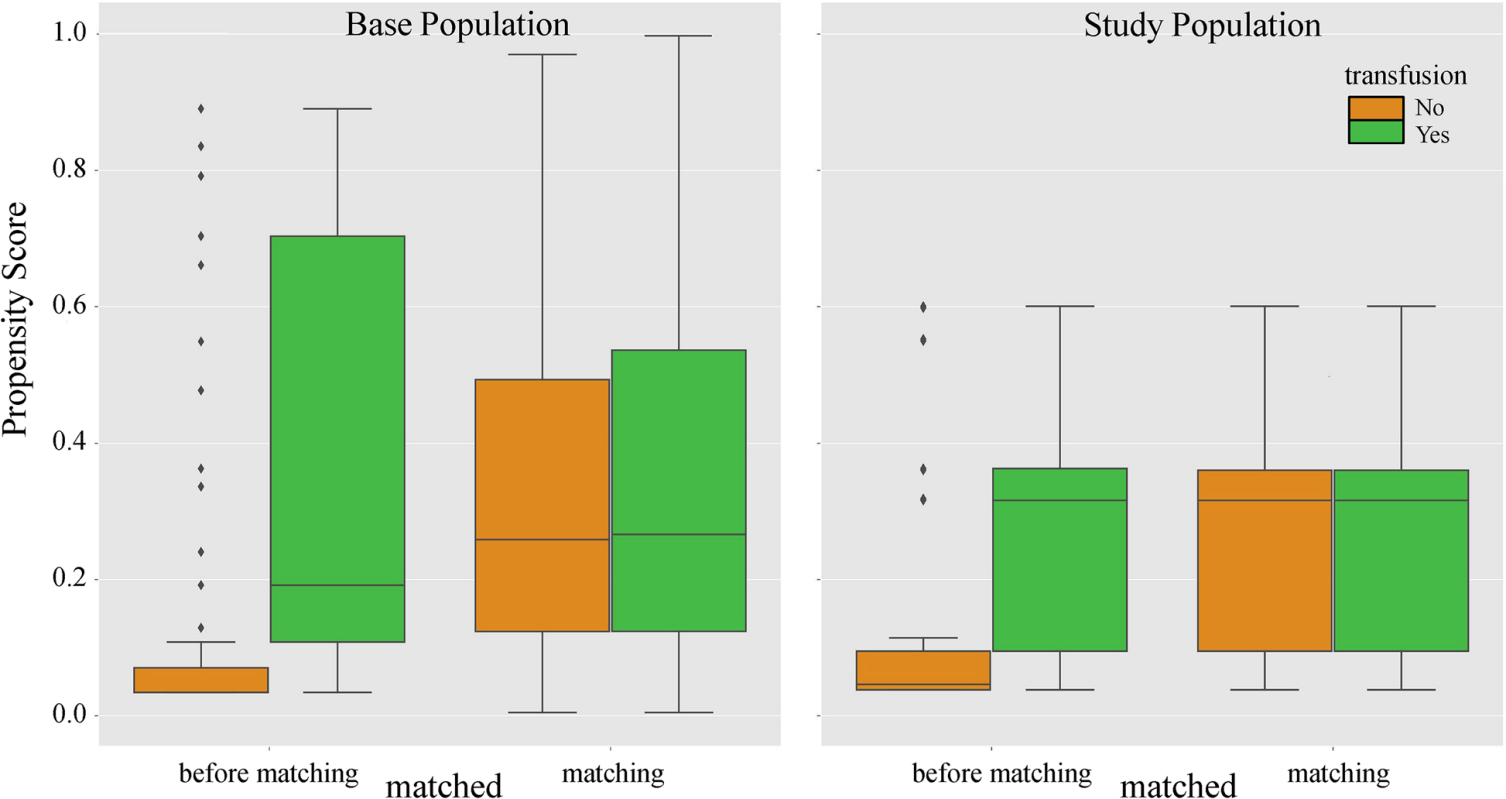
Propensity score of transfusion. ASA, American Society of Anesthesiologists; ICU, intensive care unit

In Fig. 3, we show that the overlapping range of propensity scores between comparison groups was very narrow in the base population; that is, individuals in the two groups were very heterogeneous regarding the likelihood to be transfused, and those who actually received transfusion were mostly matched to the outliers among non-transfused patients. These issues became moderated after applying the new definition of the study population, and the matched groups were more comparable regarding the propensity for transfusion.

### Study effect

**Fig. 4:**
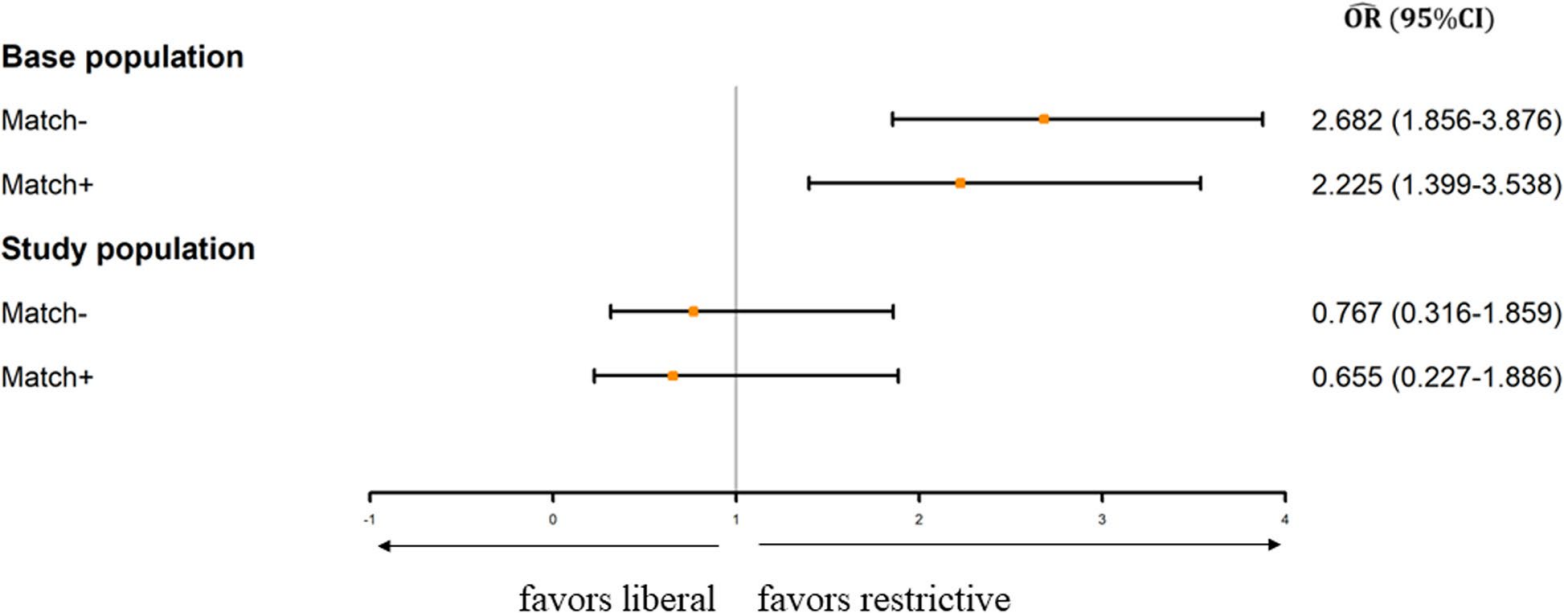
Effect estimation using different study designs. OR, odds ratio; CI, confidence interval; RCT, randomized controlled trial

We compared the estimated effect of transfusion using and not using the hemoglobin-based design. The results are depicted in Fig. 4. When estimated directly from the base population, transfusion was related to greater risk of the composite outcome, with odds ratio ($\hat{OR}$): 2.68 (95% CI: 1.86–3.86). By directly applying propensity-score matching, the estimate decreased slightly to 2.24 (95% CI: 1.43–3.49), which was still statistically significant. By contrast, the estimated effect was inverted when the new hemoglobin-based design was applied: $\hat{OR}$ (95% CI): 0.77 (0.32–1.86) and 0.66 (0.23–1.89) before and after propensity-score matching, respectively: neither was statistically significant.

### Sensitivity analysis

To demonstrate the flexibility of the hemoglobin-based design in exploring possible hemoglobin thresholds, we examined another hemoglobin range of interest (8–10 g/dL), which was higher than that of the main analysis. The $\hat{OR}$ (95% CI) was 1.87 (0.84–4.14) and 2.24 (0.69–7.27) before and after propensity-score matching, respectively (wider CI because of fewer patients with composite outcome: 67 vs. 18 before vs. after matching).

## Discussion

The development and availability of modern epidemiological and statistical methodology provide both an opportunity and challenges to obtaining valid evidence using real-world observational data, which are naturally occurring, heterogeneous, and intricate. Using this example transfusion study, we showed, step-by-step, how the choices of study population and analysis methods could affect patient heterogeneity between comparison groups as well as the ultimate study effect of interest, even leading to completely different conclusions. By using a new study design, we identified a protective role of transfusion for older patients undergoing general surgery who have a stable hemoglobin level of 7.5–9.5 g/dL. This differs from the general findings in other patient subgroups, necessitating caution when recommending a transfusion threshold for this fragile population.

In Additional file 1: Table 1, we summarize the effect estimates using our new design and those from RCTs of similar study populations. The comparison results showed good agreement, suggesting the validity of our method and demonstrating that in the transfusion field of study, RCTs and real-world studies can work in harmony to address the same clinical question. For instance, a meta-analysis including three RCTs carried out among older patients undergoing hip surgery demonstrated that liberal transfusion at a hemoglobin threshold of 10 g/dL or more could result in more favorable outcomes than more restrictive thresholds [31]. Myocardial infarction is common in older populations, and another RCT showed that patients with this condition may benefit more from liberal transfusion at a hemoglobin threshold of 10 g/dL than 8 g/dL [32]. These studies, as well as our findings, reveal the existence of a degree of anemia that patients cannot compensate themselves, without transfusion. Our focus on identifying the lowest tolerable patient anemia level is an important difference to most existing observational studies, in which patients with or without severe anemia were treated equally when relating transfusion to outcomes. Consequently, the protective role of transfusion has been overshadowed by findings showing that transfusion is harmful. Here, we enumerate a few early works that reinforce our findings. In 2001, a Medicare data-based study showed that transfusion is associated with lower short-term mortality among older patients with acute myocardial infarction if the patient’s hematocrit on admission is < 30% (equivalent to hemoglobin 10 g/dL) [33]. A similar observation was reported in 2010 from an analysis among older patients undergoing major non-cardiac surgery in Veteran’s Administration hospitals in the United States, using 30-day postoperative mortality as the study outcome [34]. A key feature of these two studies and ours, relative to other observational studies, is emphasis on the role played by measures of the degree of anemia (hemoglobin, or hematocrit) in investigating the transfusion effect.

At the other extreme, transfusion is not recommended when the hemoglobin level is above 10 g/dL [22]. According to our previous report, patients with hemoglobin greater than 10 g/dL account for nearly a quarter (23.6%) of perioperative transfusions, despite the low transfusion rate (varying from 0.2 to 10.9%) beyond this clinical standard [15]. This indicates blood overuse among non-anemic surgical patients, and inclusion of this in observational studies could place the studies at a high risk of bias regarding the transfusion–outcome association. The upper limit of 10 g/dL has implications for the study efficiency in the experimental design as well. For instance, in the latest meta-analysis on transfusion strategy [3], the transfusion rates were no more than 50% in the two comparison arms (even in the liberal transfusion arm) of several RCTs conducted without such a limit in the patient inclusion criteria. This means that at least half of patients in the two comparison groups received exactly the same intervention, i.e., no transfusion, making any comparison less likely to reveal a difference in patient outcomes. Our design is similar to RCTs designed by Carson and colleagues [32, 35] in that we apply an upper limit for patient hemoglobin; moreover, we set a lower limit that could further increase study efficiency.

In our study, the variable of mass bleeding demonstrated the largest SMD in the base population. A similar finding was reported by Yang et al. [36]. In their study, patients in the comparison groups were different regarding most baseline characteristic covariates; after propensity-score matching, nearly all covariates became well balanced (SMD < 10%), except intraoperative blood loss. This shows that bias owing to bleeding (a strong indication of transfusion) is common and may be too strong to be corrected using analytic methods alone. Besides the restriction strategy applied in this study (i.e., exclusion of patients with mass bleeding), there may be other options within the study design. For instance, in a previous study, we performed a separate analysis of cases of mass bleeding [16], and our finding has been confirmed in a growing number of RCTs dedicated to these particular cases [37].

The hemoglobin-based definition of the study population is central to our new design, and it is proven to be the most effective way to reduce patient heterogeneity. There are multiple functions of this definition. First, the stable hemoglobin range efficiently excludes active bleeding, unexplained non-transfusion with severe anemia, and irrational blood overuse, which are complex yet common situations in the real world and sources of severe patient heterogeneity related to clear indications of transfusion. Second, the definition largely retains the original authenticity of clinical practice; for instance, we included 10 patients aged 90 years and over, the inclusion of whom is ethically unfeasible in RCTs. This offers great potential to extend the study population to a different, unexplored, and broader patient spectrum. Third, the definition can be applied in prospective cohorts as well as retrospective data analyses, allowing for flexibility in exploring different thresholds within one study, as demonstrated in our main analysis and sensitivity analysis. This means greater efficiency than the trial-and-error approach adopted in RCTs that use arbitrarily selected, fixed thresholds [38]. Additionally, our approach can be useful (or at least complementary) to personalized medicine designs where a biomarker (e.g., hematocrit or hemoglobin concentration) drives the intervention decision [39]. Fourth, a far-reaching implication of the definition is that it unites the objectives of observational studies and RCTs, paving the way for an accelerated pace of evidence generation and augmentation.

In our new study design, we propose the use of SMD rather than *P* value as a measure of patient heterogeneity. This is because the sample size in an observational study is usually large (e.g., *n* = 6225 in our base population) and not predetermined, making the interpretation of *P* values problematic. By contrast, SMD is a standardized measure of difference that can be used to compare contributions to the characteristic skewness between groups. Such comparison is critical to our design because it allows investigators to identify the most important and less-important factors that determine patient heterogeneity between groups so as to devise further strategies. For instance, there were some non-key covariates (body mass index and others) that had an SMD of > 10% in Study Match+, but their impacts on the study effect can be easily controlled through the regression approach. Additionally, comparisons can be made throughout the study by carefully monitoring the changes in SMD values, which are valid regardless of the sample sizes. Owing to these valuable properties, the SMD initially proposed in 1969 (for power calculation of a *t*-test) [25] has found a popular application in meta-analyses and RCTs [40], and we expect wider application of this measure in observational studies.

Beyond the field of transfusion research, success in replicating RCT evidence using observational data have, to a large extent, bolstered confidence in the use of real-world evidence. However, our study and others show that this is not an easy task when it comes to complex medical interventions [41]. Thus, a concern of both methodological and practical importance is under what conditions a real-world study offers reliable evidence [42]. An initiative funded by the US Food and Drug Administration recently found that concordance between real-world evidence and RCTs is not guaranteed, and the selection of comparators with similar indications substantially influences result validity [41]. Our study confirmed this finding. In particular, we showed that: (1) propensity-score matching is no magic bullet, because it addresses issues of “propensity” but not systemic unmeasured bias; (2) it works only when a relatively homogenous study population is selected according to indications that are most relevant for an intervention under investigation during the design phase; (3) thus a real-world study requires both tailored design and analysis. Our method provides a pragmatic approach to systematically identifying and reducing patient heterogeneity throughout the design–analysis process, which would be valuable in real-world studies of transfusion and other complex medical interventions.

This study has several limitations. First, although over 75% of initial transfusions were administered ahead of the postoperative interval, we could not ascertain the temporality of the transfusion and patient outcomes. This issue is common in observational studies and is critical to associating outcome to transfusion. Second, our analyses were limited to inpatient complications; thus, the long-term effects of transfusion could not be assessed. Third, only a few measured confounding factors were controlled; therefore, the study cannot replicate the role of RCTs in which randomization can rule out all potential confounders (measurable or unmeasurable). Fourth, because the study population was patients with a stable hemoglobin level of 7.5–9.5 g/dL (i.e., mild anemia), the effect estimate cannot be generalized to the base population (or more general population) that includes severe, mild, and non-anemic conditions.

In conclusion, the conduct of a real-world observational study requires tailored design and analysis, and the validity of findings can be improved by using caution with respect to patient heterogeneity in real-world data. Our newly proposed study design has good potential to harmonize the effect estimation obtained from an observational design with that of an experimental design, which can contribute to exploring new areas and ultimately promoting evidence-based transfusion practice.

## Supplementary Information

**Additional file 1: Table 1.** Comparison of effect estimates from observational studies using our proposed design and randomized controlled trials.

## Data Availability

The data that support the findings of this study are available from the National Health and Family Planning Commission but restrictions apply to the availability of these data, which were used under license for the current study, and so are not publicly available. Data are however available from the corresponding authors upon reasonable request and with permission of the National Health and Family Planning Commission.

## Abbreviations

ASA: American Society of Anesthesiologists
BMI: Body mass index
CI: Confidence interval
COPD: Chronic obstructive pulmonary disease
ICU: Intensive care unit
IQR: Interquartile range
OR: Odds ratio
RCT: Randomized controlled trial
SMD: Standardized mean difference
WBC: White blood cell
