# Supplementary material for: Generating real-world evidence compatible with evidence from randomized controlled trials: a novel observational study design applicable to surgical transfusion research
Source: BMC Med Res Methodol. 2022 Dec 6;22:312. doi: 10.1186/s12874-022-01787-3 (PMC9724333; doi:10.1186/s12874-022-01787-3)
Supplement: Supplementary file 1 — Additional file 1: Table 1. Comparison of effect estimates from observational studies using our proposed design and randomized controlled trials. [file 12874_2022_1787_MOESM1_ESM.docx]

**Additional file 1: Table 1.** Comparison of effect estimates from observational studies using our proposed design and randomized controlled trials

| **Category** | **Study** | **Study population** | **Hemoglobin interval of interest** | **Outcome** | **Effect size (liberal vs restrictive)** |
| --- | --- | --- | --- | --- | --- |
| Older patients | **Present study (main analysis)** | older patients (≥60 years) undergoing general surgery | 7.5-9.5 g/dL | Death or complications | 0.66 (0.23-1.89) |
|  | Gregersen et al (2015) | older patients (≥65 years) undergoing hip surgery | 9.7-11.3 g/dL | Death or complications | 0.78 (0.56-1.10) |
|  | Carson et al (2011) | older patients (≥50 years) undergoing hip surgery | 8-10 g/dL | Death or complications | 1.11 (0.83-1.49) |
|  | Foss et al (2009) | older patients (mean age: 81 years) undergoing hip surgery | 8-10 g/dL | Death or complications | 0.08 (0.01-0.61) |
|  |  |  |  |  |  |
| Cardiac or vascular surgery patients | **Our previous report (2021)** | patients (≥18 years) undergoing cardiac, cerebral, or vascular surgery | 7-10 g/dL | Death | 0.74 (0.16-3.39) |
|  | Murphy et al (2015) | patients undergoing cardiac surgery | 7.5-9 g/dL | Death | 0.73 (0.41-1.32) |
|  | Mazer et al (2017) | patients undergoing cardiac surgery | 7.5-9.5 g/dL | Death | 1.18 (0.87-1.59) |
|  | Carson et al (2013) | patients undergoing cardiac surgery | 8-10 g/dL | Death | 0.14 (0.02-1.12) |
|  | Bracey et al (1999) | patients undergoing cardiac surgery | 8-9 g/dL | Death | 1.92 (0.49-7.69) |
|  | Bush et al (1997) | patients undergoing vascular surgery | 9-10 g/dL | Death | 1.02 (0.27-3.85) |
| Orthopaedic surgery patients | **Our previous report (2021)** | patients (≥18 years) undergoing orthopaedic, general or thoracic surgery | 7-10 g/dL | Death | 0.83 (0.36-1.94) |
|  | Gillies et al (2020) | patients with hip fracture | 7-9 g/dL | Death | 0.36 (0.03-3.85) |
|  | Grover et al (2006) | patients undergoing joint replacement surgery | 8-10 g/dL | Death | 3.03 (0.12-100.0) |
|  | Carson et al (1998) | patients undergoing hip surgery | 8-10 g/dL | Death | 1.00 (0.06-16.67) |
| Coronary heart disease patients | **Our previous report (2021)** | surgical patients with coronary heart disease | 7-10 g/dL | Death | 1.00 (0.06-16.29) |
|  | Ducrocq et al (2021) | patients with myocardial infarction | 8-10 g/dL | Death | 1.39 (0.78-2.50) |

**References**

**Wang L**, Wang Z, Huang Y, et al (2021). Expanding restrictive transfusion evidence in surgical practice: a umulticentre, prospective cohort study. Blood Transfusion. doi: 10.2450/2021.0172-21. (Our previous report)

**Gregersen M**, Borris LC, Damsgaard EM (2015). Blood transfusion and overall quality of life aMer hip fracture in frail elderly patients - the transfusion requirements in frail elderly randomized controlled trial. Journal of the American Medical Directors Association 16(9):762-6.

**Bracey AW**, Radovancevic R, Riggs SA, et al (1999). Lowering the hemoglobin threshold for transfusion in coronary artery bypass procedures: effect on patient outcome. Transfusion 39(10):1070-7.

**Bush RL**, Pevec WC, HolcroM JW (1997). A prospective, randomized trial limiting perioperative red blood cell transfusions in vascular patients. American Journal of Surgery 174(2):143-8.

**Carson JL**, Terrin ML, Barton FB, et al (1998). A pilot randomized trial comparing symptomatic vs. hemoglobin-level-driven red blood cell transfusions following hip fracture. Transfusion 38(6):522-9.

**Carson JL**, Sieber F, Cook DR, et al (2015). Liberal versus restrictive blood transfusion strategy: 3-year survival and cause of death results from the FOCUS randomised controlled trial. Lancet 385(9974):1183-9.

**Carson JL**, Brooks MM, Abbott JD, et al (2013). Liberal versus restrictive transfusion thresholds for patients with symptomatic coronary artery disease. American Heart Journal 165(6):964-71.

**Ducrocq G**, Gonzalez-Juanatey JR, Puymirat E, et al (2021). EHect of a restrictive vs liberal blood transfusion strategy on major cardiovascular events among patients with acute myocardial infarction and anemia: the REALITY randomized clinical trial. JAMA 325(6):552-60.

**Foss NB**, Kristensen MT, Jensen PS, et al (2009). The eHects of liberal versus restrictive transfusion thresholds on ambulation after hip fracture surgery. Transfusion 49(2):227-34.

**Gillies MA**, GhaHar S, Moppett IK, et al (2020). A restrictive versus liberal transfusion strategy to prevent myocardial injury in patients undergoing surgery for fractured neck of femur: a feasibility randomised trial (RESULT-NOF). British Journal of Anaesthesia 126:77-86.

**Grover M**, Talwalkar S, Casbard A, et al (2006). Silent myocardial ischaemia and haemoglobin concentration: a randomized controlled trial of transfusion strategy in lower limb arthroplasty. Vox Sanguinis 90(2):105-12.

**Mazer CD**, Whitlock RP, Fergusson DA, et al (2017). TRICS Investigators and Perioperative Anesthesia Clinical Trials Group. Restrictive or liberal red-cell transfusion for cardiac surgery. New England Journal of Medicine 377(22):2133-44.

**Murphy GJ**, Pike K, Rogers CA, et al (2015). Liberal or restrictive transfusion after cardiac surgery. New England Journal of Medicine 372:997-1008.
